# Can digital M&As promote green innovation? Evidence from manufacturing companies in China

**DOI:** 10.1371/journal.pone.0339665

**Published:** 2025-12-30

**Authors:** Xia Wang, Weina Huang

**Affiliations:** 1 Business School, University of Jinan, Jinan, China; 2 Accounting School, Guangdong University of Finance, Guangzhou, China; IU Internationale Hochschule GmbH, GERMANY

## Abstract

This paper examines whether and how digital mergers and acquisitions (hereafter, M&As) affect green innovation using a sample of Chinese manufacturing firms that have implemented digital M&As. We find that digital M&As significantly promote green innovation, with digital transformation and improved dynamic capabilities serving as the underlying mechanisms through which these effects occur. The results are robust with a battery of robustness checks. In addition, we find that digital M&As have a more significant effect on promoting green innovation among private and foreign-owned companies, large-scale companies, companies facing stronger government environmental regulations and companies in heavily polluting industries. Compared to strategic green innovation, digital M&As significantly increase the likelihood of substantive green innovation, and also significantly promote collaborative green innovation of manufacturing companies. Since promoting green innovation is not directly related to digital M&As decisions, our study mitigates the endogeneity issue existing in the current literature, providing new and robust evidence for the “Solow Paradox” from the perspective of the integration of digital resources and traditional resources.

## 1. Introduction

The effect of digital resources on green economic value is well documented in literature, yet the findings are mixed. On one hand, numerous studies reveal negative effects. Digital technologies exhibit high energy consumption and increase greenhouse gas emissions, and the “New Solow Paradox” in the digital economy is proposed [1–3]. The synthetic effect of carbon market policy and digital transformation has negative effect on green innovation [4]. On the other hand, some studies confirm the positive effects. Financial technology [5], digital transformation [6,7], digital technology [8], and digital economy [9] enhance green innovation. The contradictory results may be due to the neglect of the dynamic integration of digital resources and traditional resources. The green economic value of digital resources lies in their significant improvement of the utilization efficiency of traditional resources. Another reason may be the existence of endogeneity, as companies with strong green capabilities are more capable of developing digital resources.

In this paper, we use a sample of digital M&As of Chinese manufacturing companies to solve the above-mentioned problems. For manufacturing companies, conducting digital M&As is the most direct, rapid, and effective way to obtain digital resources [10–12]. Compared with internal research and development (R&D) or strategic alliances, digital M&As can obtain a large amount of systematic digital assets and their supporting capabilities at one time [13]. Observing whether and how manufacturing companies’ acquisition and integration of external digital resources affect their green innovation constitutes an ideal scenario for testing the economic value of digital resources, especially their green economic value. Furthermore, whether to implement digital M&As is a relatively exogenous event for green innovation. The primary purpose of implementing digital M&As is to acquire digital resources and achieve digital transformation, while promoting green innovation is not the main motivation for digital M&As.

Theoretically, digital M&As by manufacturing firms can promote green innovation. From the resource-based view [14–16], manufacturing companies can directly access digital resources from external sources in the stage of digital M&As transactions, breaking through the bottleneck of green innovation resources accumulation and the dependence on traditional green innovation paths, and promoting green innovation. Meanwhile, the dynamic integration of acquired digital resources and existing traditional resources provides conditions for advancing the digital transformation of manufacturing companies. According to the dynamic capability theory [17], when the acquired digital technologies are deeply integrated into manufacturing processes, data such as product and material data, operation planning and execution data, quality control data, and supply chain and financial data at each process node are collected in real time. With the analysis and application of these data, the company’s capabilities of opportunity perception, environmental adaptation, coordination and integration, and learning and absorption are enhanced, which will enable manufacturing companies to identify green innovation opportunities and organize resources to embrace them effectively and efficiently, thereby promoting green innovation.

We obtain data on digital M&As of manufacturing companies to empirically investigate the influence of digital M&As initiated by manufacturing companies on their green innovation. We find that digital M&As exert statistically significant positive effects on green innovation. Mechanism analysis shows that digital M&As influence green innovation by driving digital transformation and enhancing the dynamic capabilities. Then we use propensity score matching (PSM) method and two-stage least squares (2SLS) method to address the potential endogeneity issue arising from sample selection bias and reverse causality. Heterogeneity analysis shows that digital M&As have a more significant effect on promoting green innovation among private companies, large companies, companies facing stronger government environmental regulation and companies in heavily polluting industries. We construct a *Probit* model to detect the impact of digital M&As on different types of green innovation. The results show that compared to strategic innovation, digital M&As can effectively enhance substantive green innovation. We further identify sample involving joint R&D with other companies from the original green innovation data, and find that digital M&As also promoted collaborative green innovation.

This paper makes the following contributions: Firstly, it contributes the debate about the effect of digital resource on green economic value, and offers new Chinese micro-level evidence for the “Solow Paradox” [1]. As a major manufacturing nation with heavy reliance on traditional resources and a rapidly developing digital economy powerhouse, China offers an ideal empirical setting for exploring the aforementioned question. Our study takes digital M&As initiated by Chinese manufacturing companies as its research sample, empirically demonstrating how digital M&As promote the green innovation potential from the perspective of integrating digital and traditional resources. This not only enriches the contextual depth of existing research but also provides actionable insights for manufacturing companies to balance digital development and green growth.

Secondly, it contributes to the literature on digital M&As. Most existing literature focuses on the digital M&As of platform companies [18–24]. However, traditional companies constitute the main part of the market, and they differ significantly from platform companies in terms of digital infrastructure, business models, and value creation paths. Therefore, researching the digital M&As of traditional companies can fill a gap in current studies. In addition, unlike literature on green M&As (e.g., heavily polluting firms acquiring clean technologies) [25,26], this paper uniquely examines digital resource-targeted acquisitions conducted by manufacturing companies and their resulting green innovation value, thus advancing the understanding of M&As in the digital age.

Thirdly, it contributes to the literature on environment-related innovation especially the relationship between digital resources and green innovation. Environment-related innovation can be expressed using phrases such as green innovation [27–29], eco-innovation [30,31], and sustainability innovation [32–34]. Existing literature on environment-related innovation mainly focuses on the effect of government environmental regulations [35–45], and corporate internal management factors [46–49]. This paper investigates the impact of digital M&As on green innovation, expanding the relevant research literature on the factors influencing environment-related innovation. Moreover, we overcome the endogeneity issue in studies on the relationship between digital resources and green innovation by selecting manufacturing companies’ digital M&As as a relatively exogenous event.

Finally, it opens the “black box” of how digital M&As of manufacturing companies promote green innovation, and proposes that promoting digital transformation and improving dynamic capabilities are its potential mediating mechanisms. This emphasizes the importance of digital transformation and dynamic capabilities as the key roles in leveraging digital M&As to generate green innovation effects.

The remainder of this paper is structured as follows. Section 2 provides a literature review, and section 3 provides the theoretical analysis and research hypotheses. The research design is presented in section 4. Section 5 describes the empirical analysis. In section 6, we conduct further research. Conclusion is given in section 7.

## 2. Literature review

### 2.1 Economic consequences of digital M&As

This paper examines the impact of digital M&As on green innovation. Therefore, the subsequent literature review focuses on the economic implications of digital M&As and the factors influencing green innovation. The literature related to digital M&As initiated by platform companies mainly explores the motivations, monopolistic effects, and anti-monopoly measures. Platform enterprises are typical two-sided markets with network externality and user locking effect [50–53]. Most of its digital M&As are to obtain monopoly resources such as user attention and innovation ability, realize data monopoly, expand market power, and build a platform ecosystem [18–23]. To maintain their market position, large platform companies even carry out killer M&As [24]. The research on digital M&As initiated by traditional companies mainly studies their economic consequences. Implementing digital M&As is an important way for traditional companies to realize digital transformation. Digital M&As help industrial enterprises establish digital knowledge bases, which can drive digital innovation and increase enterprise value [54]. It has also been pointed out that digital M&As can promote enterprise innovation and innovation quality, and promote the upgrading of the value chain [12,55].

### 2.2 Influencing factors of green innovation

Existing literature on the influencing factors of green innovation primarily concentrates on corporate internal characteristics and external environmental regulations. Relevant research indicates that poorly governed firms produce fewer green patents, suggesting inefficient corporate governance may be a major barrier to improving environmental efficiency [46]. Green innovation strategy positively affects both exploitative and exploratory green innovation, with green supply chain integration playing a partially mediating role between them [47]. Green learning orientation positively impacts both types of green innovation, and green knowledge acquisition partially mediates this relationship [48]. Dynamic capabilities exert a significant influence on green innovation performance [49]. Green innovation requires capital investment. Studies have found that green finance can incentivize enterprises to enhance their level of green technological innovation by alleviating financing constraints [56]. Both green finance and international technology spillovers can promote green technological innovation by enhancing regional innovation capabilities [57]. Digital finance can increase the level of green technological innovation, thereby improving energy and environmental performance [58]. Conversely, political capital significantly inhibits firms’ green product and process innovation performance [59]. Corporate Social Responsibility (CSR) and Environmental, Social, and Governance (ESG) performance also affect green innovation. Research shows that CSR significantly promotes both green product innovation and green process innovation, with green dynamic capabilities acting as a mediator in this relationship [60]. Improvements in ESG performance significantly drive green innovation [61]. Additionally, corporate environmental ethics positively influences both green product innovation and green process innovation [62].

Regarding the impact of external environmental regulations on green innovation, divergent perspectives exist: one promoting and the other inhibiting such innovation. The promoting perspective is largely based on the Porter Hypothesis, which posits that environmental regulations can stimulate green technological innovation. This is because regulations can exert external pressure to drive innovation, reduce uncertainty regarding environmental investments, and increase compliance costs, thereby prompting firms to adjust their product portfolio or production processes [35–37]. Conversely, the inhibiting perspective argues that environmental regulations increase compliance costs, thereby crowding out resources for innovation and negatively impacting green innovation [38,39]. Relevant research indicates that stronger regulatory and normative pressures related to environmental issues trigger environmental innovation, particularly in firms facing larger legitimacy gaps [63]. Direct environmental regulations exert a significant incentivizing effect on green technological innovation within heavily polluting industries [41]. Furthermore, distinguishing green innovation into green process innovation and green product innovation, studies reveal that environmental regulations primarily affect financial performance through green process innovation, whereas market turbulence mainly influences financial performance through green product innovation [42].

### 2.3 Green M&As and green innovation

The literature most closely related to this paper focuses on green M&As and green innovation. Most studies suggest that green M&As significantly enhance corporate green innovation through acquiring clean technologies, complementary resources, and knowledge integration. For instance, heavy-polluting enterprises can improve green innovation levels via green M&As, with government subsidies amplifying this effect. Technology-oriented green M&As are particularly effective in stimulating green technological innovation, and environmental regulations further reinforce these incentives [26,64,65]. However, some studies present divergent perspectives. For example, Sun et al. (2024) find that the impact of green M&As on green innovation is time-sensitive: green mergers by polluting firms promote innovation within the first 1–2 years post-merger, but the effect diminishes by the third year [66]. Additionally, Yang et al. (2023) argue that green M&As primarily serve as strategic responses to legitimacy pressures (e.g., complying with government regulations or societal expectations) but fail to contribute substantively to pollution governance transition. While green M&As enable green industry switching through acquiring clean sectors, they do not improve resource utilization efficiency or reduce pollution. In contrast, efficiency-driven green innovation proves more impactful for sustainable transformation [25].

Existing literature has conducted extensive research on digital M&As and green innovation, which provides a solid literature foundation for this paper. However, there are still issues that can be further explored as follows: First, research on the economic consequences of corporate digital M&As remain insufficient. No literature has systematically examined the impact, mechanisms, and consequences of digital M&As on green innovation in manufacturing enterprises from the perspective of green innovation. Second, green innovation is a complex investment activity, and research conclusions are still controversial. Studies on the influencing factors of green innovation have mostly focused on environmental regulatory tools and internal management characteristics of enterprises. Due to the adoption of different research scenarios, perspectives, and variable measurement methods, the conclusions drawn are somewhat divergent. Third, the theoretical mechanism through which digital M&As affect green innovation remains a “black box”. There is currently no systematic research in the literature on how digital M&As influence green innovation, and the specific impact path and operational mechanism are still unclear. Based on this, this paper takes manufacturing enterprises as the research object, empirically analyzes the impact and mechanism of digital M&As on green innovation, and can effectively fill the gaps in current research.

## 3. Theoretical analysis and research hypothesis

### 3.1 Basic theories

The basic theories mainly include the resource-based view and the dynamic capability theory. The resource-based view suggests that if a firm obtains valuable, scarce, imitable, and irreplaceable resources, it will have the potential to achieve sustained competitive advantages [67,68]. According to the theoretical viewpoint, in the context of green transformation, for manufacturing companies, to build a sustainable competitive advantage need to construct a resource system for green innovation. In the digital economy era, acquiring modern digital resources to support green innovation is essential. Manufacturing companies can directly acquire digital technology resources, digital knowledge resources, and user data resources through digital M&As, thereby establishing a resource foundation for green innovation. Resources are the foundation of capabilities. Dynamic capabilities refer to the ability to develop, integrate, and restructure internal and external resources to respond to rapidly changing external environments [15,16]. Building on this definition, dynamic capabilities can be viewed as a multi-dimensional concept that can be measured more systematically. For example, dynamic capabilities can be decomposed into the capabilities to identify opportunities in the external environment and the capabilities to coordinate and adjust internal resources [17]. Furthermore, this paper combines the resource-based view, knowledge view, and organizational learning to divide dynamic capabilities into four dimensions: opportunity perception capability, environmental adaptation capability, coordination and integration capability, and learning and absorption capability [69].

### 3.2 Digital M&As and green innovation

Manufacturing companies require timely and accurate information to make informed decisions on green innovation, such as market demand information for eco-friendly products and services, input-output information on green resources in the manufacturing process, quantitative information on the impact of pollution emissions on the environment. By engaging in digital M&As, manufacturing companies can quickly and directly acquire the digital technology of the target companies. And by integrating the acquired digital technology into their manufacturing processes, they can significantly improve the availability and timeliness of information. Consequently, manufacturing companies will be able to identify market demands for green innovation more promptly and accurately than before, and can quickly allocate enterprise resources to meet this green market demand. Moreover, through digital M&As, manufacturing companies can achieve intelligent manufacturing, which will provide more possibilities for promoting green innovation.

To achieve green innovation, companies also require comprehensive knowledge resources in multiple fields such as energy conservation, carbon reduction, production and management. It is difficult to achieve green innovation breakthroughs solely based on the current knowledge in a certain field. Therefore, in the context of the digital age, establishing a digital knowledge base to enrich existing knowledge for manufacturing companies is a feasible and challenging approach [70]. Compared to internal development, digital M&As, as an external development method, can help quickly build a digital knowledge base and improve knowledge integration capabilities [54]. By implementing digital M&As, manufacturing companies can obtain innovative knowledge in the digital field. Especially because the dynamic and scalable nature of digital technology [71], it can drive knowledge combinations at the stage of post-merger integration, thereby bringing stronger green innovation effects. At the same time, manufacturing companies can use the acquired digital resources to establish a green innovation platform and collaborate with other companies in the supply chain for green innovation. This collaborative approach will facilitate the expansion of the knowledge base for green innovation and achieve greater success in this field.

The financing advantages brought by digital M&As can provide financial support for promoting green innovation. Promoting green innovation entails substantial capital investment. Due to the significant R&D risks associated with green innovation, many manufacturing companies face financing constraints on green innovation. Manufacturing companies conducting digital M&As can effectively convey crucial information about their digital transformation to the capital market, making it easier to access external financing opportunities, which can alleviate the financing constraints faced in their R&D and green innovation process, and subsequently promote green innovation.

In summary, since achieving green innovation requires the support of various resources, manufacturing companies’ implementation of digital M&As help them acquire new digital technologies and digital knowledge, and makes it easier to obtain external capital investment. Furthermore, as the post-merger integration deepens gradually, the acquired digital resources and traditional resources are successfully integrated, with traditional resources achieving higher utilization efficiency and better supporting green innovation. Therefore, the following hypothesis is proposed.

Hypothesis 1 (H1). Manufacturing companies’ implementation of digital M&As can promote green innovation.

### 3.3 Mediation mechanism of digital transformation

Digital transformation refers to the gradual process of utilizing digital technologies to optimize organizational processes and business models, thereby altering the path of value creation [72]. The majority of manufacturing companies lack the necessary resources for digital transformation, and implementing digital M&As to acquire digital resources can effectively overcome the current predicament of insufficient resources for digital transformation. The acquired digital technology can promote the generation of data, and accelerate information acquisition, storage, and processing. After the post-merger integration, the acquired digital resources are deeply integrated with manufacturing processes, and the basis for business decisions shifts from limited operational information to vast data resources [73]. Therefore, digital M&As are conducive to promoting the digital transformation of manufacturing companies.

Digital transformation can effectively promote green innovation of manufacturing companies. Firstly, digital transformation can enhance the decision-making utility of data and provide more effective data support for managers to formulate precise strategies for green innovation. For instance, after the realization of digital transformation, it is possible to interact with users in real-time to obtain timely market demand information related to green innovation. Secondly, digital transformation empowers manufacturing companies to swiftly integrate research and development resources in response to market demands for green innovation. On one hand, digital technology is utilized to improve operational processes by minimizing value loss at each process node, optimizing resource allocation efficiency, and prioritizing high-quality innovative resources for critical process nodes. This will ultimately improve the effectiveness of green innovation. On the other hand, digital technology is utilized to expand the scope for allocating innovative resources and ensure seamless participation of diverse stakeholders within the innovation ecosystem across temporal and spatial dimensions as well as interdisciplinary boundaries, thereby promoting green innovation. Therefore, the following hypothesis is proposed.

Hypothesis 2 (H2). Manufacturing companies’ implementation of digital M&As can promote green innovation by driving the digital transformation.

### 3.4 Mediation mechanism of dynamic capabilities

In the stage of digital M&As transaction, the most direct manifestation is the successful acquisition of the digital resources of the target companies. In the stage of post-merger integration, the acquired digital resources are organically integrated into the manufacturing and management processes. Acquired digital technologies can help manufacturing companies transform massive and disordered data into useful information to support decision-making [74]. Therefore, digital M&As can improve the dynamic capabilities of manufacturing companies. First of all, digital M&As enable manufacturing companies to interact with customers in a timely manner, monitor new developments in the industry in real-time, and enhance their perception of market opportunities. Secondly, with the deepening of post-merger integration, manufacturing companies gradually realize digital transformation and improved their resilience to complex environments. Thirdly, because the dynamic capabilities of integration functions are nested in organizational conventions [17], digital M&As can enable manufacturing companies to establish a collaboration and sharing mechanism across organizational boundaries, thus enhancing the ability of resource coordination and integration. Fourth, digital M&As can directly acquire the digital knowledge, digital technology and business models of the target companies, expand strategic resources and knowledge of manufacturing companies, and improve the ability to learn and absorb exogenous knowledge.

Dynamic capabilities enable manufacturing companies to achieve more green innovation. The stronger the opportunity perception capability, the more external information manufacturing companies can obtain, the more green innovation opportunities they can identify, the lower the risk of green innovation they face, and the more green innovation achievements they can achieve. Environmental adaptation capability enables manufacturing companies to take flexible countermeasures in time when facing market changes so that the green research and development goals can be matched with the changing market environment. Coordination and integration capability enables manufacturing companies to fully allocate available resources around the green research and development goals and effectively promote the process of green research and development. Learning and absorption capability enables manufacturing companies to quickly absorb innovative knowledge and technology, and generate new knowledge on the basis of existing knowledge, so as to achieve green innovation breakthroughs. In short, from the dynamic capabilities view, digital M&As enable manufacturing companies to identify more opportunities for green innovation and organize resources to embrace these opportunities effectively and efficiently. Therefore, the following hypothesis is proposed.

Hypothesis 3 (H3). Manufacturing companies’ implementation of digital M&As can promote green innovation by improving the dynamic capabilities.

**Fig 1:**
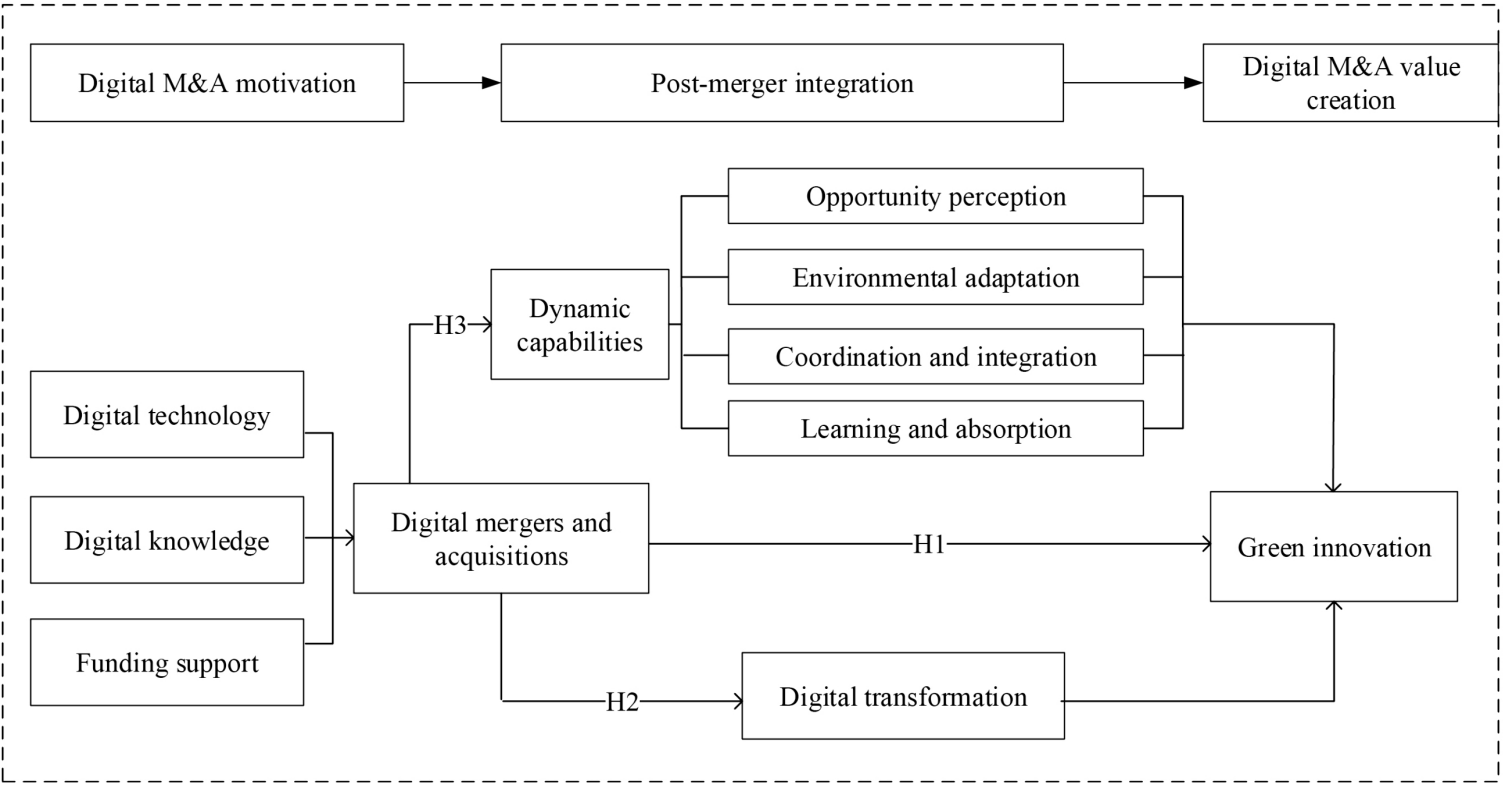
Research framework.

Fig 1 illustrates investigate framework of this paper.

## 4. Research design

### 4.1 Sample selection and data collection

This paper selects M&A events announced between 2005 and 2021, where the acquirers are manufacturing listed companies in the Shanghai and Shenzhen A-share markets, as the initial research samples. The following processing steps are conducted: (1) Considering the existence of cases where M&As are announced but not actually executed, we exclude samples of unsuccessful merger and acquisition transactions. (2) For samples with multiple M&As executed within one year, only the first merger and acquisition is retained [75]. Since the explanatory variable in this paper is a dummy variable, this processing will facilitate subsequent data collation without affecting the measurement accuracy of the explanatory variable. (3) Companies labeled as ST (Special Treatment) or PT (Particular Transfer) are excluded because they have abnormal financial conditions, unstable operations, and low data reliability, which may increase research errors and interfere with the universality of the conclusions. (4) To avoid the interference of outliers on the research conclusions, the samples are winsorized at the 1% upper and lower tails. Finally, 22,220 company-year observations are obtained.

The M&As data and control variables data of manufacturing companies are sourced from the China Stock Market & Accounting Research Database (CSMAR). The series of data related to green innovation are from the Chinese Research Data Services (CNRDS). The data on digital transformation, which is an intermediary variable, is derived from the text of annual financial reports. The basic data of dynamic capabilities, another intermediary variable, is obtained from the CNRDS database.

### 4.2 Variable definitions

#### 4.2.1 Digital M&As (*MA_Dig*).

This paper tries to investigate the relationship between digital M&As initiated by manufacturing companies and green innovation. *MA_Dig* takes the value of 1 if the manufacturing company implements digital M&As, otherwise, it takes the value of 0. Following extant literature [54,72,76], a digital merger and acquisition is defined as a merger and acquisition activity that includes the following keywords in its transaction profile:(1) Digitization, digital resources, digital assets, digital technology, digital platform, digital transformation; (2) Intelligent, artificial intelligence, intelligent manufacturing, intelligent planning, intelligent optimization, intelligent Q&A; (3) Information technology, informatization, networking, Internet, Internet of Things, big data, 5G technology; (4) Cloud computing, cloud storage, cloud platform; (5) Automatic inference, OCR, machine learning, machine vision, machine translation, deep learning, robotics, speech recognition, image recognition, neural networks, text recognition, text reading, expert system, learning algorithm, augmented reality, virtual reality, virtual community; (6) Blockchain, nanotechnology, edge computing, mobile computing, quantum computing, quantum technology, 3D printing, e-commerce.

In light of the fact that some transaction profiles may contain digital-related keywords without constituting genuine digital M&As activities, potentially in an attempt to align with national policies or investor preferences. We validated the effectiveness of the previously mentioned keyword identification methodology. We manually examined identified digital M&As transactions by reviewing whether the targets mentioned in their M&As announcements qualified as digital assets, and further verifying that these acquisitions were executed by the acquiring company to develop its own digital capabilities.

#### 4.2.2 Green innovation (*Green_inn*).

Following previous studies [77,78], *Green_inn* is represented by the cumulative count of green patent applications, including the number of green invention patent applications (*Green_Inv*) and the number of green utility model patent applications (*Green_Uma*). The reasons are as follows. First, green innovation represents the ultimate manifestation of resource utilization efficiency, and the number of patent applications as an indicator of innovation output better reflects corporate green innovation capabilities. Second, the number of patent applications provides a more accurate reflection of green innovation levels compared to the number of granted patents. The patent granting process involves greater uncertainty and instability, while patent technologies are likely to influence corporate green performance even during the application process. Therefore, patent application data proves more reliable and timely than granted patent data. Additionally, to avoid the right-skewed distribution of data, the number of patent applications is increased by 1 and then logarithmized.

#### 4.2.3 Digital transformation (*Dig_tra*).

As a summary and guiding external report, a company’s annual financial report serves as a carrier for the company to convey information to external information users. The willingness and achievements of a company in actively carrying out digital transformation are likely to be reflected in its annual report. The frequency of words related to a company’s digital transformation in the annual report is used to measure the degree of digital transformation [79]. Therefore, we set the following model to quantify digital transformation.


$${Dig\_tra}_{i,t}=Ln({AI}_{i,t}+{BC}_{i,t}+{CC}_{i,t}+{BD}_{i,t}+{DTA}_{i,t}+\text{1})$$ (1)

*i* indexes companies, and *t* indexes years. The variables in brackets of Equation (1) represent the word frequencies of artificial intelligence, blockchain, cloud computing, big data, and digital technology in the annual financial report respectively.

#### 4.2.4 Dynamic capabilities (*Dyn_cap*).

Following previous studies [69,80,81], we divide the dynamic capabilities (*Dyn_cap*) into four sub-dimensions, assign different weights to the four sub-dimensions using the entropy weight method. The calculation method for dynamic capabilities is as follows.

(1) Opportunity perception capability (*opportunity*)

Opportunity perception capability is measured by knowledge base breadth. The calculation equation is as follows.


$${Breadth}_{i,t}=\text{1}-\sum {\left(\frac{Z_{imt}}{Z_{it}}\right)}^{\text{2}}$$ (2)

*Breadth*_*i,t*_ represents the knowledge base breadth of the company *i* in year *t*, and the larger the value, the stronger the opportunity perception capability. *Z*_*imt*_ represents the cumulative number of patents filed by company *i* under group m as of year *t*; *Z*_*it*_ represents the cumulative number of patents filed by company *i* in all large groups up to the year *t*.

(2) Environmental adaptation capability (*adaptation*)

Environmental adaptation capability is measured using the coefficient of variation of the sample firms’ annual spending on three main categories: research and development, capital and advertising. To keep the direction of the coefficient of variation consistent with the adaptability, this paper takes a negative value of the coefficient of variation. The calculation formula is as follows.


Adaptation=−σ/mean (3)

In Equation (3), Adaptation indicates the environmental adaptation capability. The larger the value, the stronger the environmental adaptation capability. *σ* and *mean* represent the standard deviation and average of R&D, capital and advertising expenditures in the current year.

(3) Coordination and integration capability (*coordination*)

Coordination and integration capability is measured by asset turnover, which is the ratio of operating income to the average annual total assets.

(4) Learning and absorption capability (*absorption*)

Learning and absorption capability is measured using R&D expenditure intensity, i.e., the ratio of annual R&D expenditure to operating income of the sample firms.

(5) Dynamic capabilities

We use the entropy weighting method to assign objective weights to each sub-dimension to calculate dynamic capabilities. The calculation steps are as follows.

Step 1: Data standardization.

$$Y_{ij}=[X_{ij}-min(X_{j})]/[max(X_{j})-min(X_{j})]$$ (4)

*Y*_*ij*_ is the standardized value, *X*_*ij*_ is the indicator *j* of company *i*, max(*X*_*j*_) and min(*X*_*j*_) are the maximum and minimum values of the indicator *j* respectively.

Step 2: Find the information entropy of each indicator.

$$E_{j}=-ln(m{)}^{-\text{1}}\sum_{i=\text{1}}^{m}P_{ij}lnP_{ij}$$ (5)

In Equation (5), $P_{ij}=Y_{ij}/\sum_{i=\text{1}}^{m}Y_{ij}$, m refers to the number of companies.

Step 3: Determine indicator weights.

$$W_{j}=(\text{1}-E_{j})/(k-\sum_{j=\text{1}}^{k}E_{j})$$ (6)

In Equation (6), *k* is the number of indicators. After determining the weights of each sub-dimension by the entropy weight method, the expression of dynamic capabilities is obtained as follows.


$$Dny_{cap}=\text{0}.\text{1}\text{2}\text{7}\times opportunity+\text{0}.\text{2}\text{7}\text{4}\times adaptation+\text{0}.\text{1}\text{3}\text{8}\times coordination+\text{0}.\text{4}\text{6}\text{1}\times absorption$$ (7)

#### 4.2.5 Control variables.

The core logic for selecting control variables is as follows: these variables are inherently related to companies’ green innovation capabilities and digital M&As behaviors. Incorporating them into the model can effectively strip off the interference of irrelevant factors, ensuring that the relationship between the core explanatory variable and the explained variable is purely identified. The main control variables include company size (*Size*), asset-liability ratio (*Lev*), board size (*Board*), equity concentration (*Share*), institutional investor shareholding ratio (*Inst*), listed company age (*Age*), property right (*Soe*). The company size affects green innovation investment and digital M&As capability, the asset-liability ratio is related to financial risk and M&As financing capacity, the board size is associated with innovation decision-making efficiency, the equity concentration determines the decision-making direction of innovation and M&As, the institutional investor shareholding ratio affects green innovation and M&As decisions through governance effects, the age of listed companies reflects the differences in innovation and M&As capabilities at different stages of development, and the property right affects related behaviors due to differences in responsibilities and resource acquisition between state-owned companies and private and foreign-owned companies. In addition, controls for annual and industry-fixed effects are included. Table 1 shows the definition of the main variables.

**Table 1:** Definition of variables.

Variable	Explanation	Definition
*Green_inn*	Green innovation	The natural logarithm of the total number of green patent applications filed by a company in a given year. Calculated as: Ln(Total Green Patent Applications + 1)
*MA_Dig*	digital M&As	A dummy variable which equals 1 if a manufacturing company conducts digital M&As, 0 otherwise
*Dig_tra*	Digital transformation	Equation (1) for the calculation method
*Dyn_cap*	Dynamic capabilities	Equation (7) for the calculation method
*Size*	Company size	Natural logarithm of the company’s total assets at fiscal year-end
*Lev*	Asset-liability ratio	Calculated as: Total Liabilities/ Total Assets at fiscal year-end
*Board*	Board size	Count of all seated directors as reported in annual corporate governance reports. Includes executive, non-executive, and independent directors. Measured at fiscal year-end
*Share*	Equity concentration	Percentage of common shares held by the largest shareholder at fiscal year-end, calculated as: (Shares held by largest shareholder/ Total outstanding shares) × 100%
*Inst*	Institutional investor shareholding ratio	Aggregate percentage of shares held by qualified institutional investors (mutual funds, pension funds, insurance companies) as of fiscal year-end
*Age*	Listed company age	Years since IPO calculated as: (Observation year – IPO year) + 1
*Soe*	Property right	Dummy variable: 1 = State-owned enterprise (defined by ≥50% ultimate state ownership), 0 = Non-SOE (including private and foreign-owned firms)
*Ind*	Industry	Dummy set based on CSRC 2012 Manufacturing Subsector Classification
*Year*	Year	Year fixed effects dummy variables

### 4.3 Model setting

To test the hypothesis proposed in this paper, the following equations are designed.


$$Green\_inn_{i,t}=\alpha_{\text{0}}+\alpha_{\text{1}}MA\_Dig_{i,t}+\sum Controls+\sum Year+\sum Ind+\varepsilon_{i,t}$$ (8)

$$Dig\_tra_{i,t}/Dyn\_Cap_{i,t}=\beta_{\text{0}}+\beta_{\text{1}}MA\_Dig_{i,t}+\sum Controls+\sum Year+\sum Ind+\varepsilon_{i,t}$$ (9)

$$Green\_inn_{i,t}=\lambda_{\text{0}}+\lambda_{\text{1}}MA\_Dig_{i,t}+\lambda_{\text{2}}Dig\_tra_{i,t}/Dyn\_Cap_{i,t}+\sum Controls+\sum Year+\sum Ind+\varepsilon_{i,t}$$ (10)

In Equation (8), if *α*_*1*_ is significantly positive, it indicates that digital M&As significantly promote the green innovation of the manufacturing company, and H1 is established. If *β*_*1*_ in Equation (9) is significantly positive, and *λ*_*2*_ in Equation (10) is significantly positive, and *λ*_*1*_ is smaller than *α*_*1*_, it proves that digital transformation and dynamic capabilities are the underlying mechanisms, and H2 and H3 are established.

## 5. Empirical analysis

### 5.1 Descriptive statistics

The results of descriptive statistics of the main variables are shown in Table 2. It can be seen from Table 2 that the mean value of green innovation is 0.764, the maximum value is 4.466, and the minimum value is 0, indicating that there are significant differences in the level of green innovation among sample companies, which provides the premise for the empirical study. The mean value of digital M&As is 0.009, indicating that the proportion of digital M&As in sample companies is 0.9%, which is consistent with the fact that digital M&As are still in the initial stage of development. The mean value of the digital transformation variable is 0.042, the minimum value is 0, the maximum value is 1, and the median value is 0.009, indicating that there are significant differences in the digital transformation among sample companies. The mean value of dynamic capabilities is 0.250, the minimum value is 0.011, the maximum value is 0.415, and the median value is 0.274. In addition, the values of control variables are within a reasonable range, and there is no significant deviation in the samples.

**Table 2:** Descriptive statistics.

Variables	N	Mean	SD	Min	Median	Max
*Green_inn*	22220	0.764	1.039	0.000	0.000	4.466
*MA_Dig*	22220	0.009	0.094	0.000	0.000	1.000
*Dig_tra*	22220	0.042	0.091	0.000	0.009	0.880
*Dyn_cap*	22220	0.250	0.103	0.011	0.274	0.415
*Size*	22220	21.932	1.052	19.852	21.808	25.394
*Lev*	22220	0.404	0.184	0.056	0.402	0.868
*Board*	22220	2.145	0.156	1.792	2.197	2.565
*Share*	22220	0.339	0.134	0.091	0.321	0.717
*Inst*	22220	0.353	0.228	0.000	0.353	0.849
*Age*	22220	2.064	0.724	0.693	2.197	3.296
*Soe*	22220	0.331	0.471	0.000	0.000	1.000

### 5.2 Correlation analysis

The results of correlation analysis are shown in Table 3. The correlation coefficient between digital M&As and green innovation is 0.026 and significant at the 1% level, which indicates that digital M&As are positively correlated with green innovation. However, it is necessary to further control other factors and make regression analysis to draw appropriate conclusions. The correlation coefficient between digital M&As and digital transformation is significantly positive, which indicates that digital M&As promote digital transformation. The correlation coefficient between digital M&As and dynamic capabilities is significantly positive, and whether digital transformation and dynamic capabilities can play intermediary roles needs to be further verified by constructing regression equations. In addition, the correlation coefficients of all variables are less than 0.6, indicating that there is no serious multi-collinearity.

**Table 3:** Correlation analysis.

Variables	*Green_inn*	*MA_Dig*	*Dig_tra*	*Dyn_cap*	*Size*	*Lev*	*Board*	*Share*	*Inst*	*Age*	*Soe*
*Green_inn*	1.000										
*MA_Dig*	0.026***	1.000									
*Dig_tra*	0.039***	0.040***	1.000								
*Dyn_cap*	0.342***	0.044***	0.038***	1.000							
*Size*	0.508***	0.009	−0.061***	0.176***	1.000						
*Lev*	0.221***	−0.001	−0.031***	−0.121***	0.420***	1.000					
*Board*	0.056***	−0.021***	−0.051***	−0.115***	0.208***	0.160***	1.000				
*Share*	−0.026***	−0.023***	0.006	−0.076***	0.081***	−0.001	−0.012**	1.000			
*Inst*	0.209***	0.002	−0.024***	0.109***	0.428***	0.149***	0.137***	0.207***	1.000		
*Age*	0.186***	0.013**	−0.056***	−0.050***	0.429***	0.398***	0.130***	−0.132***	0.366***	1.000	
*Soe*	0.088***	−0.025***	−0.009	−0.203***	0.253***	0.299***	0.268***	0.165***	0.246***	0.402***	1.000

Note: *, **, *** respectively denote P < 0.1, P < 0.05, P < 0.01; the same below.

### 5.3 Regression analysis

Table 4 reports the regression results of the impact of digital M&As on green innovation and the underlying mechanisms. Column (1) is the regression result of Equation (8). The coefficient of digital M&As (*MA_Dig*) is 0.150 and significant at the 5% level, indicating that digital M&As of manufacturing companies can significantly improve their green innovation level, which supports H1.

**Table 4:** Regression analysis results.

Variables	(1)	(2)	(3)	(4)	(5)
	*Green_inn*	*Dig_tra*	*Green_inn*	*Dyn_cap*	*Green_inn*
*MA_Dig*	0.150**	0.041***	0.137**	0.014***	0.118*
	(0.022)	(0.000)	(0.037)	(0.009)	(0.066)
*Dig_tra*			0.324***		
			(0.000)		
*Dyn_cap*					2.179***
					(0.000)
*Size*	0.468***	−0.007***	0.470***	0.009***	0.448***
	(0.000)	(0.000)	(0.000)	(0.000)	(0.000)
*Lev*	0.148***	−0.006	0.150***	−0.024***	0.202***
	(0.000)	(0.228)	(0.000)	(0.000)	(0.000)
*Board*	−0.115***	−0.018***	−0.109***	0.009***	−0.134***
	(0.001)	(0.000)	(0.002)	(0.003)	(0.000)
*Share*	−0.532***	0.002	−0.532***	−0.016***	−0.497***
	(0.000)	(0.734)	(0.000)	(0.000)	(0.000)
*Inst*	0.063**	0.018***	0.057*	0.005*	0.052*
	(0.048)	(0.000)	(0.073)	(0.053)	(0.099)
*Age*	−0.081***	−0.008***	−0.078***	−0.017***	−0.044***
	(0.000)	(0.000)	(0.000)	(0.000)	(0.000)
*Soe*	0.073***	0.009***	0.070***	0.00200	0.069***
	(0.000)	(0.000)	(0.000)	(0.183)	(0.000)
*Ind*	Yes	Yes	Yes	Yes	Yes
*Year*	Yes	Yes	Yes	Yes	Yes
*Cons*	−9.794***	0.283***	−9.886***	−0.130***	−9.510***
	(0.000)	(0.000)	(0.000)	(0.000)	(0.000)
*Adj_R2*	0.354	0.037	0.356	0.462	0.376
*N*	22220	22220	22220	22220	22220

Columns (2) and (3) are the regression results of Equations (9) and (10) with digital transformation (*Dig_tra*) as the mediating variable. In Column (2), the coefficient of digital M&As (*MA_Dig*) is 0.041, significant at the 1% level, indicating that manufacturing companies engaging in digital M&As have a higher degree of digital transformation. In Column (3), the coefficient of digital transformation (*Dig_tra*) is 0.324 and significant at the 1% level, indicating that digital transformation has a positive impact on green innovation. At the same time, the coefficient of digital M&As (*MA_Dig*) in Column (3) is 0.137, which is smaller than the coefficient of digital M&As (*MA_Dig*) in Column (1), and the p-value is also increased, indicating that after the digital transformation (*Dig_tra*) variable is added, the explanatory power of digital M&As (*MA_Dig*) on green innovation has decreased. Therefore, digital transformation (*Dig_tra*) is the underlying mechanism of digital M&As promoting green innovation, which supports H2.

Columns (4) and (5) are the regression results of Equations (9) and (10) with dynamic capabilities (*Dyn_cap*) as the mediating variable. In Column (4), when *Dyn_cap* is the explained variable, the coefficient of digital M&As (*MA_Dig*) is 0.014, significant at the 1% level, indicating that the dynamic capabilities of manufacturing companies have been improved by engaging in digital M&As. In Column (5), the coefficient of *Dyn_cap* is 2.179, significant at the 1% level, indicating that the dynamic capabilities can significantly promote green innovation. At the same time, compared with Column (1), the coefficient of digital M&As (*MA_Dig*) in this column is reduced to 0.118, and the p-value is also increased, indicating that after adding the variable of *Dyn_cap*, the explanatory power of digital M&As (*MA_Dig*) on green innovation has been reduced. Consequently, digital M&As promote green innovation by improving the dynamic capabilities of manufacturing companies, thereby providing support for H3.

Although the aforementioned empirical findings confirm that digital M&As significantly promote green innovation in manufacturing companies, it is crucial to recognize the potential risks associated with digital M&As. For instance, failed technology integration may lead to a short-term decline in production efficiency, and companies might even be plunged into financial distress due to prohibitively high digital transformation costs. Cultural conflicts could undermine organizational synergy efficiency, thereby hindering the implementation of green innovation strategies. These potential negative consequences highlight the critical prerequisite for digital M&As to enhance green innovation. Only through proactive mitigation of these risks can digital M&As maximize their enhancing effects on green innovation.

### 5.4 Robustness test

#### 5.4.1 Propensity score matching (PSM) method.

To address endogeneity concerns stemming from sample selection bias, this paper reapplies the empirical tests using the propensity score matching (PSM) method. Firstly, we use the logit model to estimate the propensity score of each listed manufacturing company to conduct digital M&As. Secondly, we use the 1:1 unreverted nearest neighbor matching method to match the digital M&As samples and set the matching radius to 0.01. Table 5 lists the matching results.

**Table 5:** Sample characteristics before and after PSM matching.

Variables	Unmatched Matched	Mean value		T-value	P-value
		Treated	Control		
*Size*	U	22.027	21.976	0.62	0.538
	M	22.027	21.970	0.51	0.613
*Lev*	U	0.382	0.412	−2.17	0.030
	M	0.382	0.363	1.06	0.292
*Board*	U	2.072	2.131	−4.34	0.000
	M	2.072	2.073	−0.06	0.951
*Share*	U	0.313	0.342	−2.97	0.003
	M	0.313	0.322	−0.75	0.455
*Inst*	U	0.350	0.355	−0.28	0.779
	M	0.350	0.339	0.50	0.619
*Age*	U	2.060	2.084	−0.45	0.650
	M	2.060	1.990	1.00	0.318
*Soe*	U	0.208	0.340	−3.94	0.000
	M	0.208	0.178	0.75	0.451

The samples obtained by PSM are used to re-regress Equation (8)–Equation (10), and the results are shown in Table 6. It can be found that the regression results further support the conclusions of this paper.

**Table 6:** Regression results of PSM-matched samples.

Variables	(1)	(2)	(3)	(4)	(5)
	*Green_inn*	*Dig_tra*	*Green_inn*	*Dyn_cap*	*Green_inn*
*MA_Dig*	0.230**	0.039**	0.209**	0.024***	0.156
	(0.021)	(0.023)	(0.036)	(0.007)	(0.142)
*Dig_tra*			0.541*		
			(0.073)		
*Dyn_cap*					2.979***
					(0.000)
*Size*	0.644***	−0.023**	0.656***	0.018***	0.521***
	(0.000)	(0.014)	(0.000)	(0.000)	(0.000)
*Lev*	0.134	0.043	0.111	−0.088***	0.162
	(0.654)	(0.407)	(0.710)	(0.000)	(0.586)
*Board*	−0.204	−0.007	−0.200	−0.0170	−0.696***
	(0.439)	(0.885)	(0.445)	(0.450)	(0.010)
*Share*	−1.268***	0.133**	−1.340***	0.007	−1.221***
	(0.001)	(0.049)	(0.001)	(0.847)	(0.005)
*Inst*	−0.198	0.053	−0.227	0.028	0.133
	(0.415)	(0.200)	(0.350)	(0.231)	(0.625)
*Age*	−0.096	−0.013	−0.089	−0.029***	−0.010
	(0.235)	(0.361)	(0.270)	(0.000)	(0.903)
*Soe*	0.081	0.007	0.077	−0.007	0.048
	(0.551)	(0.760)	(0.569)	(0.543)	(0.729)
*Ind*	Yes	Yes	Yes	Yes	Yes
*Year*	Yes	Yes	Yes	Yes	Yes
*Cons*	−12.195***	0.418*	−12.421***	−0.225**	−9.696***
	(0.000)	(0.063)	(0.000)	(0.033)	(0.000)
*Adj_R2*	0.452	0.045	0.455	0.385	0.427
*N*	402	402	402	402	402

#### 5.4.2 Two-stage least squares (2SLS) method.

To address the endogeneity issue arising from reverse causality in green innovation demand driving digital M&As, this paper employs the two-stage least squares (2SLS) method. The instrumental variable selected is the digital M&As trends at the industry level (*MA_Dig_mean*). Regression results are presented in columns(1) and (2) of Table 7.

**Table 7:** Regression results of 2SLS and the time lag effect.

Variables	(1)	(2)	(3)	(4)	(5)
	*MA_Dig*	*Green_inn*	*Green_inn*_i,t + 1_	*Green_inn*_i,t + 2_	*Green_inn*_i,t + 3_
*MA_Dig*		87.381***	0.232***	0.154*	0.273***
		(0.000)	(0.002)	(0.071)	(0.005)
*MA_Dig_mean*	0.988***				
	(0.000)				
*Size*	0.002*	0.403**	0.493***	0.501***	0.501***
	(0.040)	(0.039)	(0.000)	(0.000)	(0.000)
*Lev*	−0.008*	0.684*	0.362***	0.370***	0.411***
	(0.050)	(0.075)	(0.000)	(0.000)	(0.000)
*Board*	−0.012**	0.749	−0.187***	−0.170***	−0.156***
	(0.000)	(0.484)	(0.000)	(0.000)	(0.002)
*Share*	−0.013***	0.344	−0.653***	−0.698***	−0.682***
	(0.007)	(0.927)	(0.000)	(0.000)	(0.000)
*Inst*	0.002	−0.024	0.087**	0.137***	0.188***
	(0.587)	(0.245)	(0.017)	(0.001)	(0.000)
*Age*	0.001	−0.121*	−0.174***	−0.202***	−0.233***
	(0.699)	(0.091)	(0.000)	(0.000)	(0.000)
*Soe*	−0.004**	0.289***	0.103***	0.107***	0.105***
	(0.025)	(0.000)	(0.000)	(0.000)	(0.000)
*Ind*	Yes	Yes	Yes	Yes	Yes
*Year*	Yes	Yes	Yes	Yes	Yes
*Cons*	−0.001	−10.654***	−9.573***	−9.702***	−9.596***
	(0.953)	(0.000)	(0.000)	(0.000)	(0.000)
*Adj_R2*			0.290	0.279	0.261
*N*	22220	22220	19263	16884	14665

Column (1) reports the first-stage regression results. The correlation coefficient is significantly positive at the 1% level, indicating a strong positive association between the instrumental variable and the independent variable. The F-statistic of 14.75 substantially exceeds the weak instrument critical value of 10, and the model passes the exogeneity test, confirming the validity of *MA_Dig_mean* as an instrumental variable.

Column (2) presents the second-stage regression results. The coefficient of digital M&As (*MA_Dig*) on green innovation (*Green_inn*) remains positive and statistically significant at the 1% level. Notably, the absolute magnitude of the *MA_Dig* coefficient is larger than that in the baseline regression (Table 4), suggesting that OLS estimation might underestimate the true impact when endogeneity is neglected. The 2SLS results robustly support our research hypothesis.

#### 5.4.3 Consider the time lag effect.

Since post-merger integration needs a certain period to realize synergistic value, this paper further considers the time lag of digital M&As on green innovation, and the values of the green innovation variable in *t + 1*, *t + 2*, and *t + 3* periods are substituted into the Equation (8) for regression. The results are shown in Column (3) to (5) of Table 7, which shows that the coefficients of digital M&As (*MA_Dig*) are 0.232, 0.154, and 0.273 respectively, and have passed the significance test, which further ensures the robustness of the conclusions.

#### 5.4.4 Replace proxy variables and sample intervals.

Firstly, the number of patent authorizations is selected to measure the green innovation of manufacturing companies for repeated tests. The regression result is shown in Column (1) of Table 8, and the coefficient of digital M&As (*MA_Dig*) is 0.104, which is still significantly positive at the 1% level.

**Table 8:** Regression results of replacing proxy variables and sample intervals.

Variables	(1)	(2)
	*Green_Grant*	*Green_inn*
*MA_Dig*	0.104***	0.171**
	(0.008)	(0.026)
*Size*	0.221***	0.561***
	(0.000)	(0.000)
*Lev*	−0.00200	0.294***
	(0.919)	(0.000)
*Board*	−0.095***	−0.093*
	(0.000)	(0.053)
*Share*	−0.236***	−0.466***
	(0.000)	(0.000)
*Inst*	0.0120	−0.0400
	(0.528)	(0.383)
*Age*	−0.032***	−0.081***
	(0.000)	(0.000)
*Soe*	0.042***	0.117***
	(0.000)	(0.000)
*Ind*	Yes	Yes
*Year*	Yes	Yes
*Cons*	−4.527***	−11.789***
	(0.000)	(0.000)
*Adj_R2*	0.211	0.368
*N*	22220	13223

Secondly, the samples from 2015 to 2020 are selected to be substituted into the equation for regression.The research window is strategically selected based on two key considerations. The 2015 launch of China’s “Internet Plus” initiative marked a pivotal shift that fundamentally reshaped corporate strategies in digital acquisitions. Ending the sample period in 2020 helps avoid potential contamination from the systemic market disruptions triggered by the COVID-19 pandemic. While the pandemic first emerged in late 2019, it was not until the first half of 2020 that its global spread and subsequent containment measures began to exert a significant and far-reaching impact on markets, corporate behavior, and related areas. Capping the sample period at 2020 allows for the preservation of a complete observation window following the implementation of policies such as the 2015 “Internet Plus” initiative. It also minimizes the “noise” caused by ongoing fluctuations in the post-pandemic period to the greatest extent, ensuring that the core influencing factors within the sample period remain more focused and the data more stable. The result is shown in Column (2) of Table 8, and the coefficient of digital M&As (*MA_Dig*) is significantly positive at the 5% level. The above tests further support the main conclusions of this paper, and the conclusions of this paper are robust.

## 6. Further research

### 6.1 Heterogeneity analysis

#### 6.1.1 Property rights.

Due to disparities in resource endowment and social attributes, the impact of digital M&As on green innovation varies between state-owned and private and foreign-owned companies. Firstly, compared to state-owned firms, private and foreign-owned firms have limited resource support and pursue resource economic benefits more. Merger and acquisition is an activity with high investment risk, high capital input and high time cost. The merger motivation of private and foreign-owned company is usually driven by economic benefits, so their digital M&As activities may bring higher green economic benefits. Secondly, state-owned companies shoulder greater social responsibilities and their M&As decisions are often subject to administrative interference or policy intervention. Consequently, the influence of digital M&As on green innovation may be constrained. Based on this premise, we propose that compared to state-owned manufacturing companies, the positive impact of digital M&As on green innovation is more pronounced in private and foreign-owned counterparts.

In order to examine differences in the effects of digital M&As on green innovation under different property rights, we divide the sample into two sub-groups: state-owned companies and private and foreign-owned companies; subsequently substituting them separately into Equation (8) for analysis. The results are presented in Columns (1) and (2) of Table 9. In the group comprising state-owned firms, the regression coefficient for digital M&As is not statistically significant; whereas within the private and foreign-owned companies group, it stands at 0.175 with a significance level of 5%. These findings indicate that while digital M&As have a weakened promotion effect on green innovation among state-owned entities; its positive influence is more evident among private and foreign-owned ones.

**Table 9:** Heterogeneity analysis results of property rights and company scale.

Variables	(1)	(2)	(3)	(4)
	Soe = 1	Soe = 0	Large	Small
	*Green_inn*	*Green_inn*	*Green_inn*	*Green_inn*
*MA_Dig*	0.235	0.175**	0.180*	0.094
	(0.152)	(0.030)	(0.089)	(0.199)
*Size*	0.478***	0.481***	0.598***	0.283***
	(0.000)	(0.000)	(0.000)	(0.000)
*Lev*	−0.046	0.284***	0.248***	0.099**
	(0.517)	(0.000)	(0.000)	(0.021)
*Board*	−0.190***	−0.085*	−0.034	−0.129***
	(0.004)	(0.073)	(0.533)	(0.001)
*Share*	−0.341***	−0.689***	−0.579***	−0.392***
	(0.000)	(0.000)	(0.000)	(0.000)
*Inst*	−0.064	0.173***	0.022	0.028
	(0.353)	(0.000)	(0.667)	(0.442)
*Age*	−0.168***	−0.117***	−0.130***	−0.032***
	(0.000)	(0.000)	(0.000)	(0.006)
*Soe*			0.074***	0.048**
			(0.002)	(0.018)
*Ind*	Yes	Yes	Yes	Yes
*Year*	Yes	Yes	Yes	Yes
*Cons*	−9.615***	−9.988***	−13.164***	−5.638***
	(0.000)	(0.000)	(0.000)	(0.000)
*Adj_R2*	0.430	0.297	0.388	0.129
*N*	6699	12564	11098	11098

#### 6.1.2 Company scale.

Compared with small companies, large companies have richer M&As experience, and the synergy effect brought by digital M&As is more significant. Large companies usually have stronger management ability, which can quickly realize integration after the completion of merger transaction, and give full play to the role of acquired digital resources in green innovation. At the same time, large companies also have more abundant basic green innovation resources and have a strong ability to absorb the innovative knowledge from the target company, so as to achieve more green innovation. Therefore, we argue that in large companies, digital M&As have a stronger effect on green innovation.

According to the median value of the company’s scale, the sample was divided into large and small groups for regression analysis. The results are presented in Columns (3) and (4) of Table 9. The results show that digital M&As have a significant positive impact on green innovation in the large group; while in the small group, the relationship is not obvious. This finding suggests that for larger companies, digital M&As can promote green innovation more significantly.

#### 6.1.3 Heavy polluting industry.

In the heavily polluting industry, manufacturing companies face intensified pressure for green transformation, thereby highlighting their motivation to engage in digital M&As activities to drive green innovation. Against the backdrop of economic and social green transformation as well as low-carbon development, there is an increasingly urgent demand for manufacturing companies in the heavy-polluting industry to mitigate pollution and reduce carbon emissions. The most fundamental way to achieve green transformation is to promote green innovation. In the process of industrial digitization, digital M&As provide a chance for heavy-polluting companies to achieve green innovation through deep integration of digitization and greening. Therefore, for manufacturing companies in the heavily polluting industry, the impact of digital M&As on green innovation is more significant.

Based on the Environmental Disclosure Guidelines for Listed Companies (promulgated by China’s Ministry of Environmental Protection in 2010) and the Guidelines for Industry Classification of Listed Companies (issued by the China Securities Regulatory Commission in 2012), the following sectors are identified as heavy-polluting industries: textiles, leather, paper, petroleum, chemicals, and metals. The samples are grouped according to whether they are in heavy polluting industries and then substituted into Equation (8) for regression.

The regression results are shown in Columns (1) to (2) of Table 10. The regression coefficient of the heavy-polluting industry group is significantly positive, while the non-heavy-polluting industry group is not significant. These results indicate that when manufacturing companies are in heavily polluting industries, digital M&As can bring more green innovation.

**Table 10:** Heterogeneity analysis results of industry and environmental regulation strength.

Variables	(1)	(2)	(3)	(4)
	Heavy polluting	Non-heavy polluting	Stronger	Weaker
	*Green_inn*	*Green_inn*	*Green_inn*	*Green_inn*
*MA_Dig*	0.280*	0.123	0.079	0.283***
	(0.097)	(0.125)	(0.356)	(0.007)
*Size*	0.391***	0.550***	0.524***	0.405***
	(0.000)	(0.000)	(0.000)	(0.000)
*Lev*	−0.249***	0.259***	0.216***	0.069
	(0.000)	(0.000)	(0.000)	(0.194)
*Board*	0.159***	−0.159***	−0.048	−0.164***
	(0.010)	(0.001)	(0.341)	(0.001)
*Share*	−0.130	−0.665***	−0.637***	−0.375***
	(0.121)	(0.000)	(0.000)	(0.000)
*Inst*	0.261***	0.140***	−0.012	0.142***
	(0.000)	(0.001)	(0.792)	(0.001)
*Age*	−0.078***	−0.105***	−0.093***	−0.074***
	(0.000)	(0.000)	(0.000)	(0.000)
*Soe*	0.098***	0.055**	0.059**	0.105***
	(0.000)	(0.014)	(0.016)	(0.000)
*Ind*	Yes	Yes	Yes	Yes
*Year*	Yes	Yes	Yes	Yes
*Cons*	−8.631***	−11.420***	−11.159***	−8.355***
	(0.000)	(0.000)	(0.000)	(0.000)
*Adj_R2*	0.329	0.396	0.377	0.323
*N*	5818	13445	11265	10531

#### 6.1.4 Government environmental regulation strength.

Environmental regulations refer to a series of policy measures taken by the government to restrict the economic activities of companies to reduce pollution emissions. Environmental regulations include command regulation and incentive regulation. Command regulations are formulated and implemented by relevant departments, which supervise the emission situation of companies and impose penalties on companies that exceed the emission standards. Incentive regulations encourage companies to voluntarily protect the environment through incentive measures such as tax reductions, subsidies, emissions trading, and priority procurement.

Under stronger environmental regulation intensity, manufacturing companies are compelled to allocate substantial resources to meet stringent environmental standards and compliance requirements, such as purchasing pollution control equipment and obtaining environmental management system certifications. These compliance costs consume a considerable amount of capital and manpower, thereby crowding out the resources that could be used for green innovation following digital M&As. Conversely, under weaker environmental regulation regimes, the more lenient policy environment grants companies greater autonomy and flexibility in innovation. Freed from stringent compliance obligations, firms can strategically integrate digital technologies with green innovation to develop new green products, processes, and services. The reduced institutional constraints amplify the synergistic effects of digital-greening integration, thereby making the promoting effect of digital M&As on green innovation more prominent.

Referring to the previous paper [82], the proportion of word count in the government work reports for each province serves as a proxy variable for environmental regulations. The vocabulary related to environmental protection includes environmental protection, environmental pollution, energy consumption, emission reduction, ecological protection, low-carbon air, chemical oxygen demand, sulfur dioxide, carbon dioxide, pm10, pm25. The samples are divided into two groups based on the median of the intensity of environmental regulations and regression is conducted respectively. The results are shown in columns (3) and (4) of Table 10. When the intensity of environmental regulations faced by manufacturing companies is stronger, the regression coefficient of digital M&As is 0.079, but it does not pass the significance test. When the intensity of environmental regulations faced by manufacturing companies is weaker, the regression coefficient of digital M&As is 0.283, which is significant at the 1% level. The regression results support the above theoretical analysis.

### 6.2 Digital M&As and substantial green innovation

To investigate the impact of digital M&As conducted by manufacturing companies on green innovation in more detail, this paper categorizes types of green innovation as substantial and strategic. As a capital-intensive investment project with prolonged time consumption and high risk, successful M&As can redefine a company’s operational boundaries, generate economies of scale benefits, and fundamentally enhance operational efficiency. By implementing digital M&As, manufacturing companies aim to break free from traditional development modes and overcome path dependence in their growth trajectory. The objective behind executing digital M&As is to achieve substantial progress towards green development. Consequently, compared to strategic green innovation, digital M&As exhibit a more pronounced influence on substantial green innovation.

Following extant research methods [77,78], substantial green innovation is represented by whether there is a green patent application for invention, and strategic green innovation is represented by whether there is a green patent application for a utility model.

The following Probit model is designed to verify the above theoretical derivation.


$$Probit\left[P\left({Green\_D}_{i,t}=\text{1}\right)\right]=\alpha_{\text{0}}+\alpha_{\text{1}}MA\_Dig_{i,t}+\sum Controls+\sum Year+\sum Ind+\varepsilon_{i,t}\;$$ (11)

In Equation (11), Green_D is a dummy variable, representing substantial innovation (*Green_D_Inv*) and strategic innovation (*Green_D_Uma*) respectively. When there is a green patent application for invention, *Green_D_Inv* takes 1, otherwise it takes 0; When there is a green patent application for a utility model, *Green_D_Uma* takes 1, otherwise it takes 0.

The results of the regression of the Equation (11) are listed in Columns (1) and (2) of Table 11. The regression result of Column (1) shows that the regression coefficient of digital M&As (*MA_Dig*) is 0.212 and significant at the 1% level, while the regression coefficient of digital M&As (*MA_Dig*) in Column (2) is not significant, indicating that compared with strategic innovation, digital M&As conducted by manufacturing companies significantly increases the possibility of substantial innovation.

**Table 11:** Digital M&As and green innovation types.

Variables	(1)	(2)	(3)
	*Green_D_Inv*	*Green_D_Uma*	*Green_Uni*
*MA_Dig*	0.212**	0.154	0.568**
	(0.028)	(0.109)	(0.032)
*Size*	0.446***	0.427***	0.784***
	(0.000)	(0.000)	(0.000)
*Lev*	0.0370	0.214***	−0.013
	(0.533)	(0.000)	(0.934)
*Board*	−0.001	−0.024	−0.539***
	(0.978)	(0.643)	(0.000)
*Share*	−0.555***	−0.353***	−1.325***
	(0.000)	(0.000)	(0.000)
*Inst*	0.114**	−0.052	−0.284**
	(0.020)	(0.284)	(0.027)
*Age*	−0.092***	−0.043**	−0.296***
	(0.000)	(0.010)	(0.000)
*Soe*	0.160***	0.030	−0.002
	(0.000)	(0.234)	(0.976)
*Ind*	Yes	Yes	Yes
*Year*	Yes	Yes	Yes
*Cons*	−11.618***	−11.192***	−14.571***
	(0.000)	(0.000)	(0.000)
*Adj_R2*	0.193	0.200	0.048
*N*	22220	22220	22220

### 6.3 digital M&As and collaborative green innovation

The growing demand for green innovation in the economy and society will drive a new trend of collaborative green innovation between manufacturing companies and stakeholders in the supply chain. On the one hand, successful digital M&As deals can enhance the collaborative green innovation capabilities of manufacturing companies, thereby strengthening their competitive edge in innovation and obtaining more opportunities to cooperate with other stakeholders. On the other hand, successful digital M&A deals are conducive to the construction of a green innovation ecological platform, which can attract more stakeholders to participate in cross-temporal and cross-regional collaborative green innovation. China has large industrial clusters have been formed in regions such as the Yangtze River Delta and the Pearl River Delta, where upstream and downstream companies in the supply chain are highly concentrated. At the same time, supply chain usually takes large companies as the core, with a large number of small and medium-sized suppliers clustering around, forming a close collaborative network. Digital M&As may promote the green innovation of the entire supply chain by integrating resources and technologies within it. Under such a system, the collaborative green innovation after digital M&As can rely on the advantages of industrial clusters to achieve resource sharing and rapid diffusion of technologies. Therefore, the implementation of digital M&As by manufacturing companies can promote collaborative green innovation.

The total number of collaborative green patent applications is increased by 1 and the logarithm is taken as the proxy variable of collaborative green innovation (*Green_Uni*), and this variable is introduced into the regression analysis. The regression results shown in Column (3) of Table 11 show that the regression coefficient of digital M&As (*MA_Dig*) is 0.568, which is statistically significant at 5%. This indicates that manufacturing companies can effectively promote the realization of collaborative green innovation through digital M&As.

## 7. Conclusion

In this paper, we use a sample of digital M&As of Chinese manufacturing companies to examine whether and how digital M&As affect green innovation. We find out that digital M&As can promote green innovation. Driving digital transformation and improving dynamic capabilities are the potential mechanisms of digital M&As affecting green innovation. M&As decisions made by private and foreign-owned companies are usually guided by economic benefits, so their digital M&As activities produce higher green innovation benefits. Due to more M&As experience, stronger management ability, and richer basic green innovation resources, the impact of digital M&As on green innovation will be more obvious in large companies. Faced with stronger pressure of green transformation, heavy pollution manufacturing companies have stronger green motivation when executing digital M&As. Digital M&As can significantly promote the substantial green innovation and collaborative green innovation of manufacturing companies. Digital M&A decisions are mainly related to acquiring digital resources and achieving digital transformation, and have no direct connection with promoting green innovation. Thus, for green innovation, digital M&A is a relatively exogenous event, which makes our study provide robust evidence for the controversies in the current literature. Meanwhile, since manufacturing companies’ implementation of digital M&As allows for good observation of the integration of digital resources and traditional resources, our study can offer new empirical insights into the “Solow Paradox”.

Based on the above conclusions, the following suggestions are proposed. For managers, first of all, they should proactively conduct digital M&As based on their own strategic needs. By implementing a digital M&A strategy, acquirers can quickly obtain the valuable digital resources of target companies, effectively break through existing knowledge accumulation barriers, and provide support for green innovation and transformation.Secondly, after the completion of digital M&As, companies should achieve in-depth integration of the acquired digital resources and their original traditional resources through successful post-merger integration. For policymakers, the first is to facilitate the digital transformation of heavily polluting manufacturing companies through digital M&As, thereby promoting green innovation and industrial green upgrading; the second is to direct resources towards green innovative products and services, such as guiding social capital to support companies in achieving green innovation through government-guided funds, thus enhancing the efficiency of green resource allocation; Additionally, differentiated policies should be formulated based on the diverse development stages and models of manufacturing companies, and establish platforms for exchanging green information, foster multi-party collaboration models for green initiatives, and facilitate collaborative efforts towards promoting green innovation.

Digital M&As may have negative effects on green innovation. As a strategic investment, digital M&As require significant capital input and take a long time. If M&A integration fails, enterprises will not only be unable to achieve the synergistic effect of green innovation, but may also weaken their original green innovation capabilities due to resource dispersion; in extreme cases, it may even lead enterprises to fall into financial distress and be forced to discontinue their green R&D projects. Paying attention to these negative effects helps manufacturing enterprises manage and control the risks of digital M&As. Additionally, the impact of digital M&As by manufacturing enterprises on green innovation varies across different economies. In developed economies with mature digital technology ecosystems, the acquired digital resources can be quickly converted into green innovation outcomes. In contrast, emerging economies generally lag behind developed economies in the independent R&D and in-depth application of digital technologies, which may prolong the time required to absorb the acquired technologies.

This study focuses on the impact and mechanisms of digital mergers and acquisitions on green innovation in Chinese manufacturing companies. While addressing gaps in existing literature, the research still has limitations. Future studies could extend in the following directions. First, explore the green value created by digital M&As in non-manufacturing sectors. Second, distinguish the heterogeneous effects of cross-border digital M&As and domestic digital M&As on green innovation. Third, non-listed small and medium-sized enterprises (SMEs) can serve as research objects to investigate the disparities in the impact of digital mergers and acquisitions on green innovation between listed companies and non-listed SMEs.

## Supporting information

S1 Data Data set. (XLSX)
